# Slab tearing and segmented subduction termination driven by transform tectonics

**DOI:** 10.1126/sciadv.ady8347

**Published:** 2025-09-24

**Authors:** Brandon Shuck, Brian Boston, Suzanne M. Carbotte, Shuoshuo Han, Anne Bécel, Nathaniel C. Miller, J. Pablo Canales, Jesse Hutchinson, Reid Merrill, Jeffrey Beeson, Pinar Gurun, Geena Littel, Mladen R. Nedimović, Genevieve Savard, Harold Tobin

**Affiliations:** ^1^ Louisiana State University, Baton Rouge, LA, USA.; ^2^ Lamont-Doherty Earth Observatory, Columbia University, Palisades, NY, USA.; ^3^ Auburn University, Auburn, AL, USA.; ^4^ Institute for Geophysics, University of Texas at Austin, Austin, TX, USA.; ^5^ Woods Hole Coastal and Marine Science Center, U.S. Geological Survey, Woods Hole, MA, USA.; ^6^ Woods Hole Oceanographic Institution, Woods Hole, MA, USA.; ^7^ Ocean Networks Canada, Victoria, BC, Canada.; ^8^ Ideon Technologies, Richmond, BC, Canada.; ^9^ Oregon State University, Corvallis, OR, USA.; ^10^ Pacific Marine Environmental Laboratory, National Oceanic and Atmospheric Administration, Newport, OR, USA.; ^11^ Dalhousie University, Halifax, NS B3H 4R2, Canada.; ^12^ University of British Columbia, Victoria, BC, Canada.; ^13^ University of Geneva, Geneva, Switzerland.; ^14^ University of Washington, Seattle, WA, USA.

## Abstract

Subduction may terminate when a mid-ocean ridge approaches a trench, introducing buoyant lithosphere that resists subduction, leading to slab detachment and plate boundary reconfiguration. Yet, the spatial and temporal dynamics of slab tearing remain enigmatic due to a lack of modern examples. Here, we integrate new seismic images with regional seismicity to investigate an actively fragmenting subduction system at northern Cascadia’s ridge-trench-fault triple junction where subduction termination is imminent. Our analyses reveal a broad shear-zone initiated at ~4 Ma by exploiting ridge-parallel fabrics of nascent oceanic lithosphere and progressively localized into a mature trench-perpendicular transform boundary. This process severed an oceanic microplate and enabled its diminished subduction relative to adjacent subducting lithosphere. Downdip, we image trench-parallel slab tears offset by the transform, suggesting lateral tear propagation was intersected by the transform boundary, facilitating efficient decoupling of the microplate while allowing adjacent subduction to continue. We propose a 4D model where transform boundaries drive laterally diachronous slab fragmentation and subduction termination.

## Teaser

Transform faults act as tectonic segmentation boundaries, enabling diachronous slab detachment and subduction termination.

## INTRODUCTION

The termination of subduction is inevitable to maintain plate tectonics over time, yet the dynamics of this fundamental process remains poorly understood (*1*, *2*). Since slab pull is an order of magnitude greater than other tectonic forces (*3*), the successful extinction of subduction requires a major “tectonic accident” to overcome this force and trigger plate reorganization. There are two main proposed scenarios for subduction termination, when subduction ceases along a convergent tectonic boundary. The first occurs when the underthrusting plate hosts thick unsubductable lithosphere such as continental blocks and oceanic plateaus that jam subduction (*1*, *4*), causing slab detachment from the buoyant obstruction and long-term change from subduction to collision with suturing. Alternatively, as a mid-ocean ridge on the incoming plate approaches the trench, the young oceanic lithosphere is very weak and positively buoyant (*5*), and hence both resist underthrusting and may be unable to sustain slab pull forces. Thus, ridge-trench impingement does not involve orogenesis but instead the colder and denser slab detaches from warmer and weaker incoming lithosphere, resulting in slab pull reduction and subduction ceasing as the tectonic engine diminishes (*6*, *7*). In both cases, slab detachment causes a decrease in tectonic driving forces and subsequent surface effects such as slab-window volcanism and topographic uplift as the underlying asthenosphere upwells to fill the void (*8*–*10*), and termination is accompanied by a plate boundary reconfiguration.

Here, we focus on the latter scenario where encroachment of a mid-ocean ridge leads to slab breakoff and transition from a subduction to transform plate boundary. The best-known example of this event is the Cenozoic termination of Farallon plate subduction beneath western North America (*11*). Plate reconstructions show that the Pacific (PAC)–Farallon ridge collided with the trench in the Oligocene and created two tectonic triple junctions migrating in opposite directions, with the expanding zone between them accommodating new right-lateral transform motion between the PAC and North American (NA) plates (*12*, *13*). This sequence is preserved by magnetic anomalies revealing numerous oceanic microplates representing fragmented pieces of the Farallon plate that were captured by the PAC plate as subduction terminated (*14*, *15*). Characteristic slab-window volcanism is preserved onshore (*16*, *17*); however, abandoned ridge segments offshore argue that the slab window cannot be explained by ridge subduction (*18*–*20*) but instead require plate tearing in young oceanic lithosphere landward of ridge axes (*6*, *21*, *22*). Whether the slab detached rapidly in a continuous along-strike fashion or instead occurred in a discrete piecewise style remains unknown. The precise spatial and temporal evolution of oceanic plate fragmentation, slab tearing, and triple junction migration associated with Farallon subduction termination and other global events remain unresolved because these events are ephemeral and poorly preserved in the geologic record. Remnant slab fragments are difficult to image with geophysical data because they may disappear into the deeper mantle, and shallowly underplated fragments thermally equilibrate with the mantle over time and their kinematic context is missing. Modern examples of ongoing subduction termination are needed to understand these dynamics.

We obtain constraints on slab breakoff and subduction termination by investigating northern Cascadia, a region that is actively undergoing this process and has a well-known tectonic history, and where new deep-penetrating seismic reflection images illuminate the subsurface structure of this archetypal ridge-trench-fault (RTF) triple junction setting. In RTF triple junction settings, a spreading ridge intersects a trench and oceanic lithosphere on one side is consumed by subduction, whereas the other side experiences translation relative to the overriding plate. Here, young oceanic lithosphere immediately adjacent to the ridge axis is exceptionally warm, buoyant, and weak, and is therefore suitable to accommodate slab tearing and breakoff. To better understand the evolution of deformation within the incoming oceanic lithosphere and slab during subduction termination, we synthesize deep-penetrating seismic images with regional earthquake catalogs along the northern Cascadia RTF triple junction. We find evidence for pervasive and widespread shearing that gradually localized into a mature trench-perpendicular transform boundary within the incoming oceanic lithosphere, forming a microplate and accommodating along-strike decoupling and subduction deceleration. Landward of the trench, we show that the microplate’s slab is actively detaching in a steep trench-parallel fault zone that is laterally bounded by vertical tearing along the trench-perpendicular transform. Meanwhile, the adjacent broader oceanic plate, across from the microplate and transform boundary, is continuing to subduct relatively undisturbed. These rare in situ constraints support a piecewise subduction termination framework where transform faults act as key segmentation boundaries that facilitate plate fragmentation, laterally diachronous slab detachment, and discrete triple junction jumps that progressively shorten subduction margins over time.

### Northern Cascadia tectonic setting

**Fig. 1.**
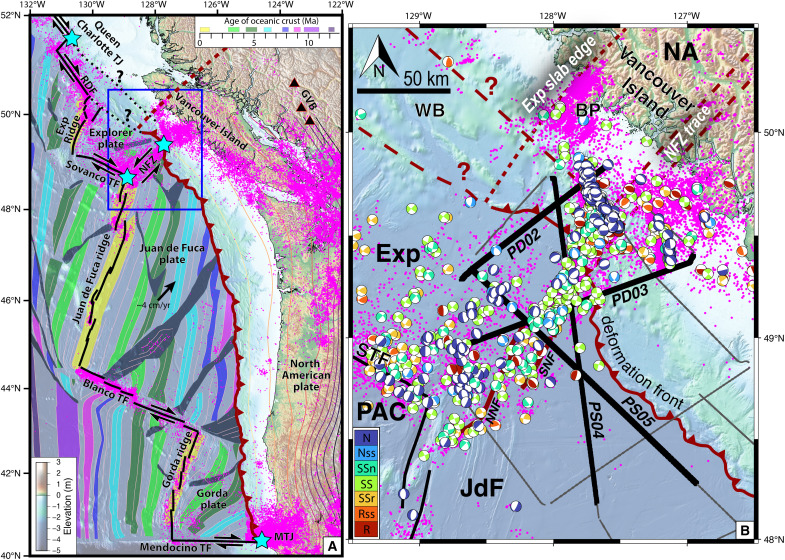
Tectonic setting of the northern Cascadia subduction zone. (**A**) Tectonic structure of the Cascadia margin and subducting oceanic plates. Thick black lines show primary tectonic boundaries and triple junctions are marked with cyan stars. The deformation front is marked by a red hatched line. Seismicity (magenta dots) is a compilation from refs (*47*, *83*–*85*). Subducting slab structure from Slab2 (*86*) is denoted with colored contours every 10 km. The dashed red line represents the northernmost edge of the subducting slab, from (*38*). Black triangles denote major volcanoes of the Garibaldi Volcanic Belt (GVB), the northern segment of the Cascade Arc. Thin light pink lines represent magnetic isochrons and their corresponding polygons are colored by age, whereas dark gray regions outline propagator wakes (*87*). Half-headed black arrows show relative motion across plate boundaries, while the large black arrow shows JdF’s convergence direction relative to a fixed North American plate from MORVEL (*88*). The blue rectangle outlines the study area shown in (B). NFZ, Nootka Fault Zone; RDF, Revere-Dellwood Fault; MTJ, Mendocino Triple Junction. (**B**) Northern Cascadia study area showing location of CASIE21 seismic profiles and regional seismicity. Earthquake epicenters (magenta) from (*40*–*43*), and focal mechanisms colored by their slip style are from (*40*, *46*). Thick red lines show mapped surface traces of the NFZ (*30*). Thin gray lines show CASIE21 seismic profiles, while thick black lines represent profile extents shown in Fig. 3. Exp, Explorer Plate; JdF, Juan de Fuca Plate; NA, North American Plate; PAC, Pacific Plate; STF, Sovanco Transform Fault; NNF, Northern Nootka Fault; SNF, Southern Nootka Fault; BP, Brooks Peninsula; WB, Winona Basin. Focal mechanism types: N, normal; Nss, normal/strike-slip; SSn, strike-slip/normal; SS, strike-slip; SSr, strike-slip/reverse; Rss, reverse/strike-slip; R, reverse.

**Fig. 2.**
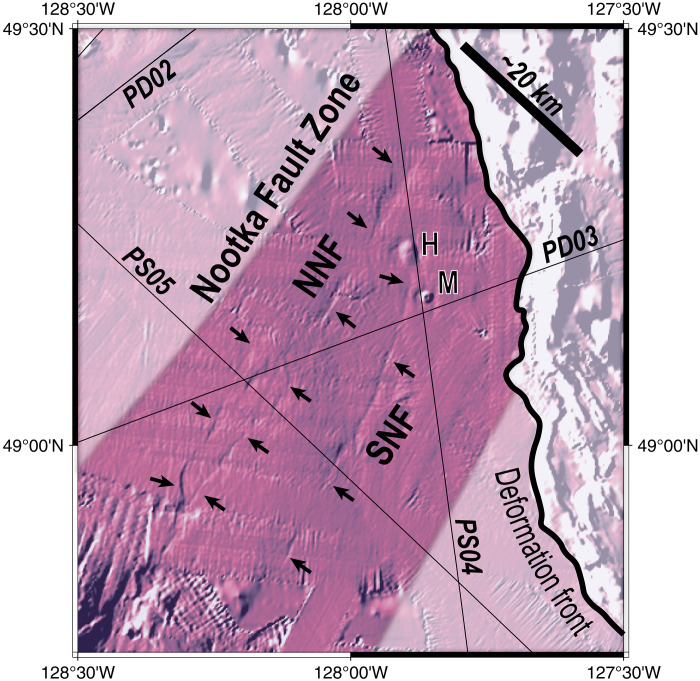
Seafloor expression of the NFZ in northern Cascadia. Map of seafloor scarps associated with active Nootka Fault Zone (NFZ) deformation. Color shading represents the gradient of seafloor bathymetry calculated from an azimuth of 300° clockwise from north using the Global Multi Resolution Topography version 4 grid (*89*). The scarps of the Northern Nootka Fault (NNF) and Southern Nootka Fault (SNF) have an average trend of ~N28°E. H, Haggis Mound; M, Maquinna Mound. Note that the NFZ is wider in the SW and narrows toward the deformation front in the NE.

Along northern Cascadia, the Explorer (Exp) and Juan de Fuca (JdF) ridges, Cascadia subduction zone, and Queen Charlotte Fault meet in a complex RTF triple junction setting offshore Vancouver Island, Canada (*23*–*25*) (Fig. 1). The Queen Charlotte Fault accommodates right-lateral strike-slip between the PAC and NA plates, while the Cascadia subduction zone hosts subduction of the JdF and Exp oceanic plates beneath North America. Kinematic plate reconstructions demonstrate that the modern Exp, JdF, and Gorda plates were previously a contiguous oceanic plate and a single RTF triple junction was stable near northern Vancouver Island since >10 million years (Ma) (*23*, *24*, *26*, *27*). However, at ~4 Ma, perhaps due to a clockwise rotation in relative PAC-NA motions (*15*, *28*) and subduction resistance beneath NA (*29*, *30*), the northernmost ridge segment jumped and/or migrated rapidly westward forming the independent Exp ridge and Exp microplate (*26*, *31*, *32*). Convergence between Exp and NA gradually slowed relative to adjacent JdF, which induced the complex Nootka Fault Zone (NFZ) that accommodates left-lateral shear between Exp and JdF, extending southwest (SW)–northeast (NE) from the northern tip of the JdF ridge to the subduction deformation front (*30*, *33*–*35*) (Figs. 1 and 2). Thus, the present-day setting may involve three triple junctions where the four plates (PAC, NA, Exp, and JdF) meet within ~300 km of each other. Modern geodetic data clearly show that convergence between Exp and NA (~2 cm/year) is markedly reduced relative to the adjacent JdF (>4 cm/year) (*36*, *37*); however, it remains unknown if the Exp slab is detached from its updip unsubducted lithosphere despite recent passive-seismic imaging in the region (*38*–*43*).

**Fig. 3.**
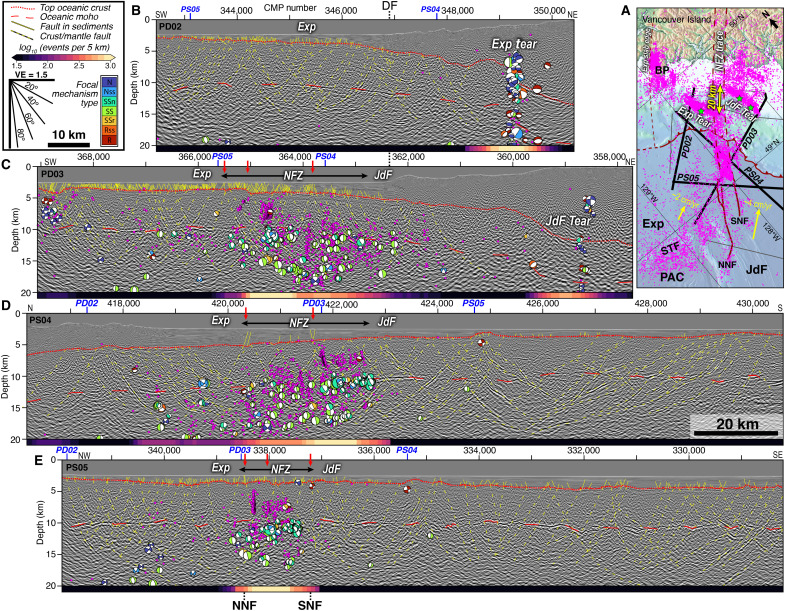
Seismic reflection images of the NFZ and subducting Exp and JdF slabs. (**A**) Oblique-view map showing seismic profile locations, earthquake epicenters (*40*–*43*), and left-laterally offset seismicity lineations coincident with imaged slab tears. Green stars show locations of *M*_w_ > 6 earthquakes that ruptured the slab tears in the last two decades. Yellow arrows show convergence vectors of the Exp and JdF plates relative to a fixed NA plate (*34*, *88*). (**B** to **E**) Interpreted PSDM MCS reflection profiles from the CASIE21 dataset. All profiles are shown at the same horizontal and vertical scales with a vertical exaggeration of 1.5. Red arrows denote profile crossings of seafloor scarps of the NNF and SNF fault strands (Fig. 2). Profile line crossings labeled in blue. Earthquake hypocenters (pink) and focal mechanisms colored by slip style are from (*40*) and projected ±15 km to profiles. Colored bars along the bottom of profiles show earthquake density in rolling 5-km bins (Materials and Methods).

**Fig. 4.**
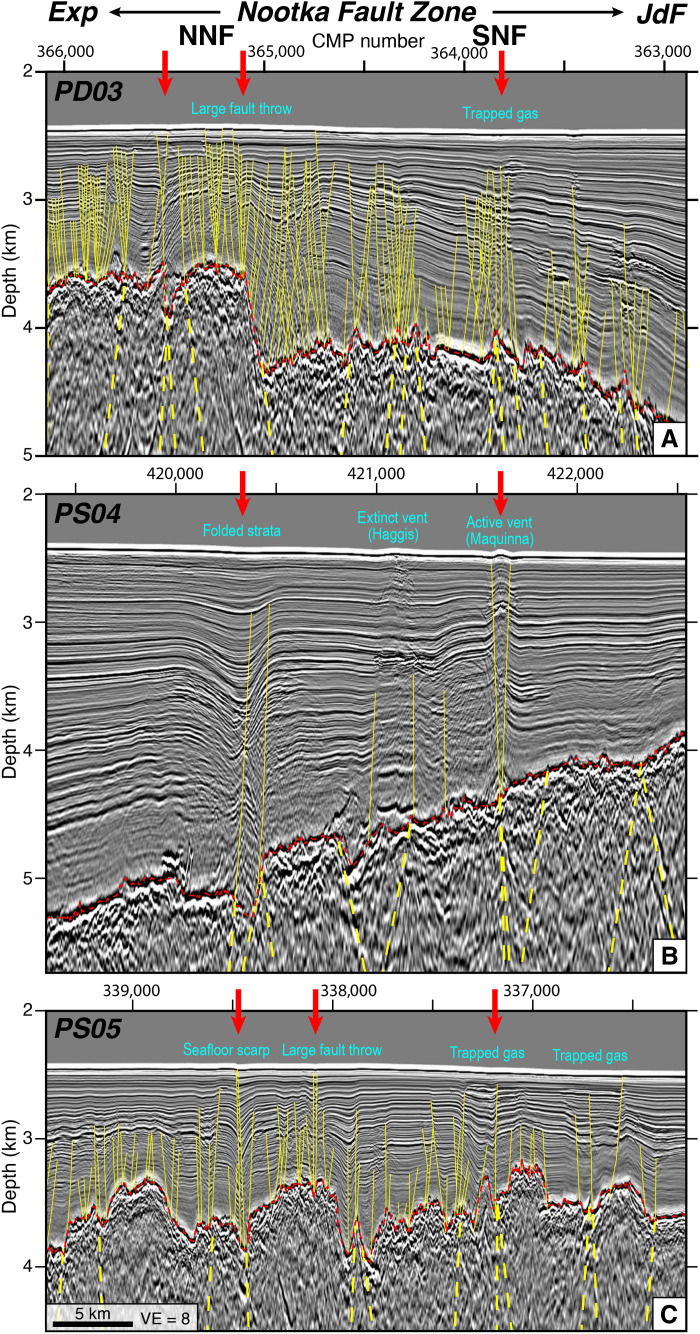
Detailed seismic images of active deformation within the modern NFZ. (**A** to **C**) Zoom-ins on PSDM seismic reflection profiles crossing the NFZ. All images are shown at the same scales with a vertical exaggeration of 8. Red arrows denote profile crossings of mapped seafloor scarps of the NNF and SNF fault strands (Fig. 2). (A) Profile PD03. The sedimentary section is heavily faulted on Exp. (B) Profile PS04. Sedimentary faults are limited on this profile and deformation appears localized on the NNF and SNF faults. (C) Profile PS05. Oceanic crust appears rougher on this profile, yet fault deformation in the sediment cover appears mostly localized on the NNF and SNF strands.

**Fig. 5.**
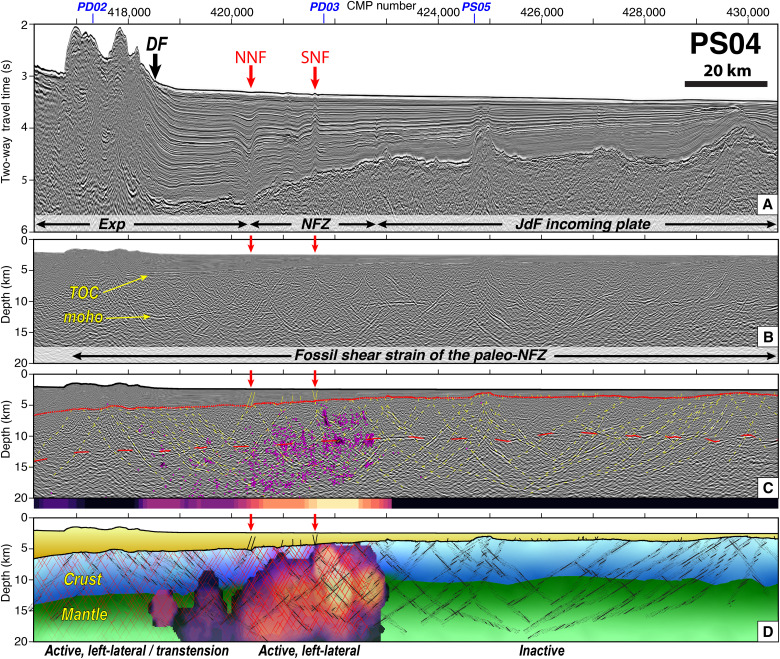
Seismic reflection image and projected seismicity on profile PS04 reveals the progressive localization of the NFZ shear zone through time. (**A**) Uninterpreted PSDM seismic reflection image converted back to two-way travel time to highlight shallow structures. The panel highlights the lack of active faults in the sediment section, except at the NNF and SNF strands of the NFZ. (**B**) Uninterpreted prestack depth migration image highlighting the unusually bright fault-plane reflectivity transecting the crust and upper mantle, which are interpreted as fossil anastomosing shear fabrics of the paleo-NFZ. (**C**) Interpreted prestack depth migration reflection image with mapped horizons, faults, and earthquake hypocenters from (*40*), projected ±15 km into the profile, are shown in pink. The bottom thin panel along the base of the section shows earthquake density as in Fig. 3. (**D**) Schematic tectonic interpretation of PS04. The early stages of subduction resistance caused formation of the paleo-NFZ, which consisted of widespread reactivation and deepening of abyssal hill faults with a left-lateral shear sense, illustrated by black hatched lines. Over time, the NFZ localized into its present configuration, where deformation has ceased on the JdF side, focused left-lateral strain is accommodated in the narrow active NFZ, and the Exp side experiences transtensional stress. The red lines schematically illustrate active faulting within the NFZ and within the Exp microplate. Colored overlay shows earthquake density with similar colors as in (C). [(B) to (D)] are shown at VE = 1.5.

**Fig. 6.**
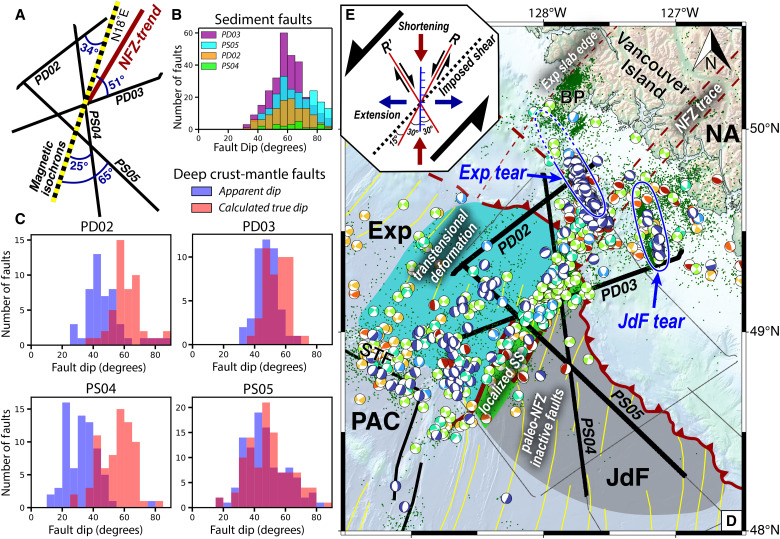
Analysis of paleo- and modern-NFZ faults within the incoming oceanic lithosphere. (**A**) Orientation of CASIE21 profiles in this study with respect to the dominant trend of magnetic isochrons of ~N18°E, and the NFZ which trends ~N28°E, as defined by seafloor scarps shown in Fig. 2. (**B**) Apparent dip distributions for faults in the sedimentary column, which are normally distributed around an average dip of ~60° on all profiles. Fault density is highest on PD03 and lowest on PS04. (**C**) Apparent dip (semitransparent blue) and inferred true dip (semitransparent red) distributions for crust and mantle fault-plane reflections on the four profiles (see Materials and Methods). For select faults within the active NFZ, we estimate true fault strikes of ~N27°E, which is nearly identical with the NFZ trend mapped by multibeam bathymetry. (**D**) Interpreted tectonic domains based on deformation history of the NFZ imaged in this study. Pervasively sheared JdF lithosphere (gray region) represents fossil deformation associated with the paleo-NFZ. Deformation of the paleo-NFZ extends over a >100-km-wide zone (gray, green, and blue regions) centered on the modern NFZ. At present, left-lateral strike-slip deformation is localized to a ~20-km-wide zone in the modern NFZ (green region). Stress concentrations within the isolated Exp microplate cause ongoing transtensional deformation as its slab gradually detaches and subduction terminates (teal region). The Exp tear is inferred (dashed blue line) as extending to its northern slab edge near Brooks Peninsula. (**E**) Schematic diagram of idealistic fault orientations and their predicted slip type for a typical left-lateral shear zone. A 10° counterclockwise orientation of modern NFZ faults from the imposed shear direction is consistent with expected left-lateral Riedel Shears (R), while the Exp and JdF tears oriented between ~45° to 75° counterclockwise from imposed shear direction are optimal to accommodate extension and normal faulting from down-dip slab bending forces.

**Fig. 7.**
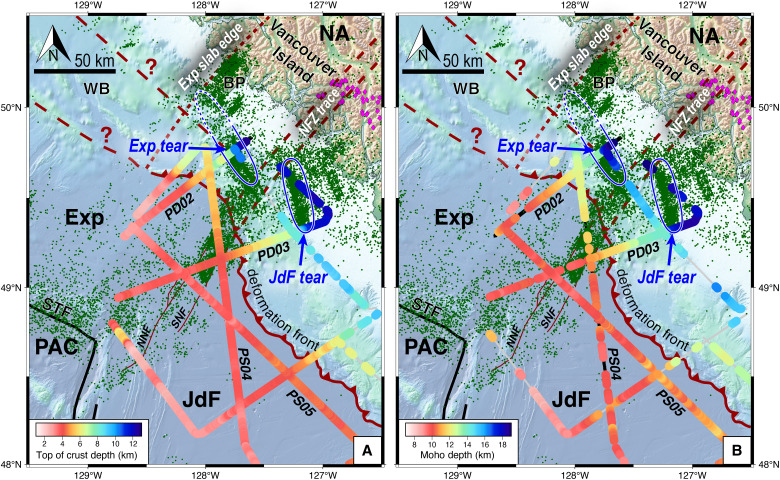
Map view of imaged slab structure and seismicity in the northern Vancouver Island region. Note the clear alignment between a drop in the top of subducting crust and Moho depths on PD02 and PD03 across earthquake lineations denoting the Exp and JdF slab tears, respectively (blue outlines). The Exp tear is inferred (dashed blue line) as extending to its northern slab edge near Brooks Peninsula. Earthquake epicenters from (*40*–*43*) are shown in dark green. Low-frequency earthquakes from (*90*) are shown in pink diamonds; note that they terminate abruptly at the projected NFZ trace, suggesting Exp slab decoupling. (**A**) Depth to top of crust horizon along CASIE21 profiles. (**B**) Depth to oceanic Moho along CASIE21 profiles.

**Fig. 8.**
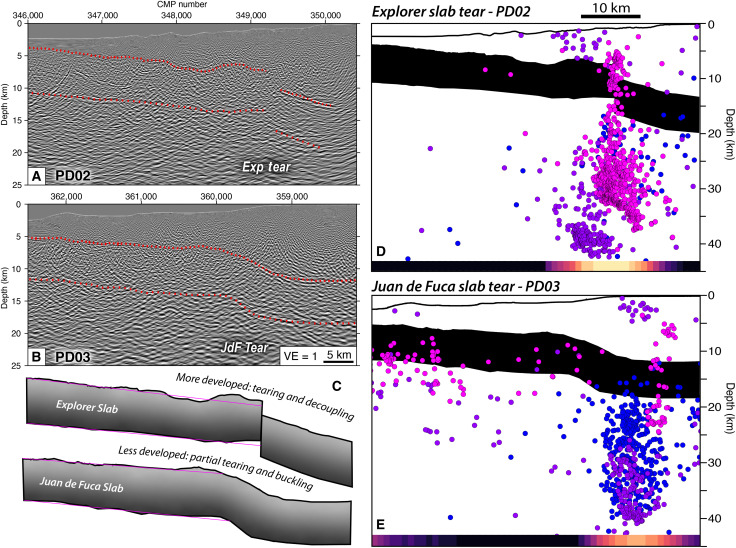
Zoom-in seismic reflection images and seismicity associated with the Exp and JdF slab tears. (**A**) Interpreted slab structure on PD02 revealing a sharp >3 km vertical offset of the slab over a short distance of ~2 km. (**B**) Interpreted slab structure on PD03. The slab here exhibits a buckled morphology with bending over a lateral distance of ~10 km. (**C**) Comparison of Exp and JdF slab geometries beneath Vancouver Island. Pink lines show linear dip trends based on slab trajectories beneath the outer accretionary prism. Note the upward flexure of the top of crust and Moho updip of the tears, and steepening downdip. The Exp tear is sharper and has enhanced local rebound in the updip direction and foundering downdip of the tear, suggesting a more mature stage of detachment. The ongoing seismicity indicates that the Exp tear is not fully detached but rather in late stages of development with pervasive focused deformation. (**D** and **E**) Interpreted slab structure and seismicity associated with the Exp/JdF tears, respectively, shown at VE = 1. Hypocenters are colored the same as in Fig. 7, and the earthquake density is the same as in Fig. 3. In (E), the primary band of seismicity locates slightly downdip of the apex of the slab buckle, which may be due to an oblique orientation of the seismic profile that crossed the southernmost tip of the tear as outlined by the seismicity lineation.

**Fig. 9.**
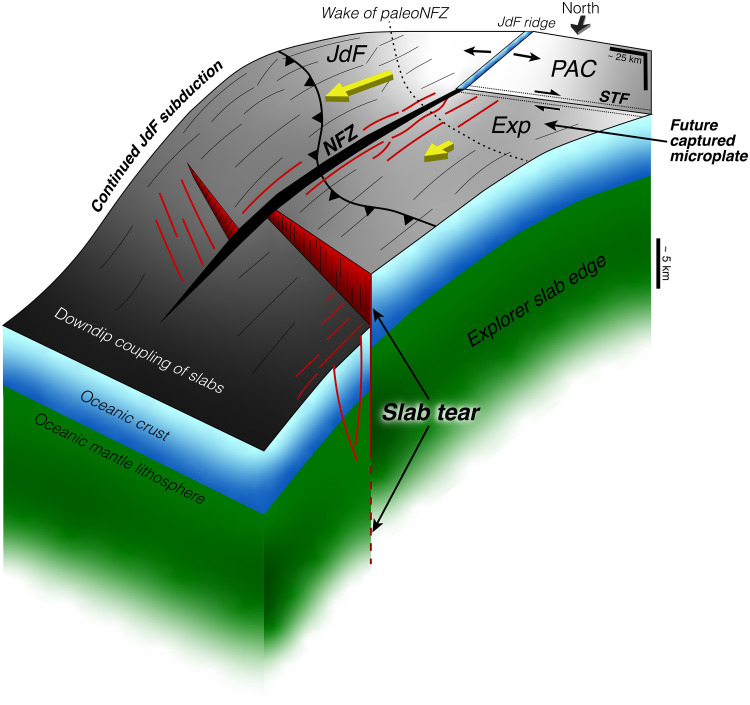
Slab fragmentation and cessation of subduction enabled by the NFZ. Schematic 3D interpretation of the NFZ-Exp-JdF triple junction region in northern Cascadia where subduction termination is imminent. The paleo-NFZ (thin black lines) developed as a broad shear zone in nascent oceanic lithosphere and reactivated inherited abyssal hill faults formed at the ridge. The weakened lithosphere buckled as a trench-parallel slab tear formed at the northern edge of the Exp slab and migrated across the paleo-NFZ wake. Recent strain localization along the modern NFZ at ~1 Ma dissected the migrating slab tear and effectively isolated the Exp microplate, leading to enhanced tearing, decoupling, and a local reduction in the slab pull force. The NFZ thus serves as a trench-normal segmentation boundary enabling piecewise slab fragmentation and subduction termination.

To investigate potential slab tearing during subduction termination, we integrate new deep-penetrating multichannel seismic (MCS) reflection data from the 2021 Cascadia Seismic Imaging Experiment (CASIE21) (*44*, *45*) with regional seismicity and focal mechanisms (*40*–*43*, *46*) to characterize tectonic deformation within the incoming oceanic lithosphere and subducting slab. The high-quality prestack depth migrated (PSDM) seismic reflection images, with colocated high-resolution hypocenters and focal mechanisms from local onshore and offshore passive seismic campaigns (Materials and Methods) offer an unprecedented glimpse into the lithospheric structure of a setting where subduction termination is ongoing. We focus on four 2D CASIE21 MCS profiles (PD02, PD03, PS04, and PS05) that transect the JdF and Exp plates, the NFZ between them (Figs. 2 to 6), and their respective slabs beneath the northern Cascadia forearc and Vancouver Island shelf (Figs. 7 to 9 and figs. S1 to S4).

## RESULTS

### Deformation of incoming oceanic lithosphere

Seaward of the deformation front, the MCS images and earthquake hypocenters reveal the active NFZ is a ~20-km-wide network of pervasive faults extending from the shallow sedimentary cover and penetrating through the crust into the upper mantle (Figs. 3 and 4). Faults in the sediment section are identified by offset sediment horizons, while deeper faults in the crust and mantle are interpreted from bright fault-plane reflectors. Deformation is largely concentrated on several NE-SW fault strands that bound the NFZ, the northern Nootka Fault (NNF) and southern Nootka Fault (SNF), which correspond with seafloor scarps mapped in bathymetry data (Fig. 2) (*30*, *35*, *47*). The NNF is bifurcated and has several en-echelon stepover faults in the SW but appears to focus to a single strand toward the deformation front. On PD03, the NNF displays large basement throw >1 km with apparent normal sense and is clearly imaged from the seafloor to 20 km depth (Figs. 3C and 4A and fig. S2). Seismicity in the NFZ is abundant and extends from the top of oceanic crust to ~10 km beneath the oceanic Moho, and focal mechanisms indicate dominantly left-lateral and transtensional strike-slip faulting (Fig. 3 and figs. S2 to S4). Within the NFZ, the Moho reflection weakens or disappears where earthquakes are concentrated, which is consistent with deep faulting and hydrothermal fluid alteration suggested by previous studies (*30*, *39*, *42*, *48*, *49*) (Figs. 3 and 5 and figs. S2 to S4). In the overlying sediments, we also find numerous seismic anomalies marked by reduced frequency waveforms capped by high-amplitude, reverse-polarity reflections (Figs. 3 and 4). We interpret these disturbances as evidence for fluid expulsion and the presence of methane gas in the stratigraphic column consistent with prior high-resolution seismic studies and submersible dives that found warm fluids and methane seeping from the Maquinna mound along the SNF (*30*, *48*) (Fig. 2).

Bounding the NFZ, the deep-penetrating images reveal an anastomosing network of bright, dipping reflections within the crust and mantle, extending to ~20 km depth and across a broad >100-km-wide zone (Figs. 3 and 5). Within the active NFZ, many of the dipping reflections link offsets in the sediments and other dipping reflections, indicating they are fault-plane reflections. Outside the NFZ on the JdF side, these deep faults do not extend into the young (<1.7 Ma) sedimentary cover (*50*, *51*), implying deformation has ceased, consistent with a lack of observed seismicity (Fig. 3 and figs. S3 and S4). In contrast, on the Exp side, extensive faulting dissects the sediment section and seismicity indicates ongoing deformation here (*52*).

The deep fault-plane reflections across the broad zone centered on the active NFZ have variable amplitude and apparent dip angles between the various seismic profiles. We carried out a geometric analysis of the mapped faults to understand their tectonic origin (Fig. 6). Several primary fault strands of the NFZ could be correlated between multiple profiles because of their obvious structural characteristics and closely spaced line crossings, which allow us to estimate their true orientations. For example, we calculate that the NNF has a true strike of N27°E (Materials and Methods), which agrees very well with the NNF strike of N28°E measured from multibeam bathymetry data of its seafloor scarp. The NNF and SNF orientations are subparallel to magnetic isochrons and are consistent with Riedel Shears predicted in a left-lateral shear zone (*30*). Extending this routine to mapped faults outside of the NFZ shows that they dominantly dip ~60° and similarly strike subparallel to magnetic isochrons (Materials and Methods; Fig. 6). This inferred orientation is further supported by variations in reflectivity between the profiles, which are brightest where apparent dips are shallowest.

On the basis of our analysis, it is likely that these faults originated as ridge-parallel abyssal hill normal faults in nascent oceanic crust and were reactivated and deepened to accommodate left-lateral transtensional shearing during subduction resistance. The widespread faulting and anastomosing pattern suggest a very weak rheology where it is easier to reactivate an adjacent inherited structure rather than continue long-lived slip on a single fault plane. The lack of large apparent throw of the top of crust and Moho horizons, especially outside of the active NFZ, suggests dominantly strike-slip displacements took place on these faults. Collectively, these observations suggest that when the NFZ began at ~4 Ma (*30*), referred to hereafter as the “paleo-NFZ,” it initiated as a wide shear-zone in extremely young oceanic lithosphere but progressively localized into the present-day configuration (Figs. 5 and 6). Hence, the modern NFZ is a unique transform boundary that segments incoming oceanic lithosphere and accommodates differential motion between the adjacent Exp-JdF plates. Since the paleo-NFZ formed at ~4 Ma and was not blanketed by impermeable sediments until <1 Ma, the paleo-NFZ would have been open conduits to facilitate extensive deep fluid circulation and hydrothermal alteration. We speculate that the unusual high-amplitude nature of the paleo-NFZ faulting is related to extensive fluid alteration within this well-connected >100-km-wide shear zone in young, warm, and weak oceanic lithosphere, and this intense fluid alteration within the faults causes a strong seismic impedance contrast with adjacent lithologies (Fig. 5).

### Tears in the subducting slab

Profiles PD02 and PD03 extend ~50 km landward of the deformation front and parallel the NFZ on the Exp and JdF sides, respectively, allowing us to characterize deformation in the oceanic slab and interactions with the NFZ as it subducts (Fig. 3). The JdF and Exp slabs underthrust the outer accretionary prism at a shallow (~3° to 6°) angle. However, at ~30 and 40 km past the deformation front on the Exp and JdF sides, respectively, both slabs exhibit a ~5 km landward drop in the top of the crust and Moho (Figs. 3, 7, and 8). The drop in the Exp slab is an abrupt break, while the JdF step-down appears as a monoclinic fold over ~10 km. These slab drops coincide with distinct ~35-km-long, trench-parallel earthquake lineations that are offset by ~20 km in a left-lateral sense across the subducting NFZ trace (Figs. 3, 7, and 8 and figs. S1 and S2). Both earthquake lineations extend from the slab tops to ~40 km depth, have ruptured in moment magnitude (*M*_w_) > 6 events in the last two decades, and exhibit dominantly normal-faulting focal mechanisms (*39*, *42*) (Figs. 2 and 4). These seismicity patterns have been thought to mark tears in the subducting slab (*39*, *40*, *42*, *43*, *53*), but previous passive-source studies lacked high-resolution imaging to resolve the slab structure across these features.

Our constraints on slab morphology revealing sharp vertical deflections coincident with steep extensional faulting at the Exp and JdF earthquake lineaments are consistent with active slab tears at different stages of maturity (Fig. 8). The abrupt break in the Exp slab with more abundant and focused seismicity is evidence of a well-developed tear in its underthrust slab. Projected earthquake hypocenters align in excellent agreement with the seismically imaged Exp slab drop and form a steep “wall” of seismicity from the slab top to ~40 km depth, indicating a mature, but not yet complete, stage of slab breakoff. Conversely, the gradual buckling of the JdF slab with more distributed seismicity reflects a less mature stage of tearing. Projected hypocenters on PD03 tend to locate slightly downdip from the apex of the monoclinic fold; however, this may be partially due to an oblique orientation crossing the southernmost tip of the seismicity lineation. Both Exp and JdF slabs exhibit an upward flexure just updip of their respective tears and a steepened plate downdip (Fig. 8), which is characteristic of dynamic rebound from local weakening and reduction in slab pull shown by geodynamic models (*6*, *9*, *54*) and geologic observations (*21*, *55*–*57*). Normal faulting earthquakes defining the Exp and JdF tears terminate at their respective intersections with the subducted NFZ (Figs. 1B and 3A), which consists of left-lateral and transtensional strike-slip events past the deformation front that diminish near its junction with the Exp tear.

## DISCUSSION

### NFZ transform–driven plate fragmentation and slab detachment

We suggest that the broad zone of pervasive faulting we image in the incoming oceanic lithosphere reflects fossil strain of the paleo-NFZ in response to early subduction resistance. The nature of these faults implies the paleo-NFZ reactivated inherited spreading-center parallel structures of nascent oceanic lithosphere immediately after its creation at the ridge. This interpretation, along with fluid-rock interactions and strain localization of the NFZ through time, has been noted by prior studies and is further validated by our data (*30*, *40*, *49*). These studies could not, however, image the deep structure of paleo-NFZ crust and mantle. Our observations of unusually high-amplitude fault-plane reflections redefine the extent of faulting in the paleo- and modern-NFZ and provide evidence of extensive hydrothermal circulation through a well-connected, >100-km-wide fault network (Materials and Methods; Figs. 5 and 6).

A persistent basal sediment nonconformity of >1 Myr found by ODP168 indicates that the paleo-NFZ crust did not have an impermeable sedimentary cover until <1.7 Ma, and therefore, paleo-NFZ faults would have been open conduits to host deep seawater percolation. Pervasive fluid alteration in open fault conduits of the paleo-NFZ is the most likely explanation for strong seismic impedance contrasts leading to bright fault-plane reflectors within the crust and upper mantle. Continued faulting and vigorous hydrothermal circulation would have dramatically reduced lithospheric strength and promoted strain localization, thus enabling concentrated left-lateral shear within the modern NFZ and deceleration of Exp beneath NA relative to adjacent JdF. Strain localization at the NFZ also concentrates transtensional stress within the Exp microplate as it rotates clockwise and gradually couples to the PAC plate (*26*, *29*, *34*), which is supported by ongoing distributed internal deformation and curved magnetic isochrons (*29*, *41*). In contrast, paleo-NFZ faulting on the JdF side was abandoned after NFZ strain localization, because the weak NFZ decouples the adjacent JdF oceanic plate and allows for continued subduction beneath North America.

The unprecedented seismic reflection images, active seismicity, and tectonic context of slab tearing in northern Cascadia offers a rare opportunity to observe both trench-parallel and trench-perpendicular slab tears and their interactions leading to subduction termination. Downdip of the deformation front, the seismic data image two major trench-parallel tears in the downgoing slab that are offset ~20 km left-laterally by the NFZ (Figs. 3, 7, and 8). The geometry and orientation of the NFZ-mirrored Exp and JdF tears suggest that they were perhaps initially a single continuous tear that was later disrupted by left-lateral transform motion. Given the average Exp-JdF motion deficit of ~20 mm/year (*30*, *31*, *34*–*36*), the 20 km NFZ-related offset in the slab tears could have been achieved in ~1 Myr. This timing agrees well with our independent estimate of NFZ strain localization, as the unfaulted stratigraphy blanketing the JdF in the paleo-NFZ zone is almost entirely younger than 0.90 Ma (*50*, *51*). Although the tears were likely once a continuous, trench-parallel tear, the Exp tear appears more mature than the JdF tear, evidenced by its deeper and steeper slab geometry, a larger and sharper slab offset, and more focused seismicity outlining a near-vertical 2D plane through the apex of the slab offset (Fig. 8). Hence, the present-day structure is best explained by a major trench-parallel tear that initiated before NFZ localization and propagated laterally to the southeast through weakened lithosphere of the paleo-NFZ wake. Subsequently, once strain localized along the NFZ, dissection of the laterally migrating trench-parallel tear by trench-normal tearing along the modern NFZ transform offset the tear into two distinct segments (Fig. 9).

Additional observations suggest the Exp microplate is partially decoupled and subduction cessation is imminent. The northern edge of Exp slab representing continuous subduction since the Miocene is oriented NE-SW near Brooks Peninsula, as defined by heat flow and geodetic data, onshore fault and topography patterns, the limit of arc volcanism in British Columbia, and seismic tomography (*27*, *36*–*38*, *58*). Between the subducted NFZ trace and Exp slab edge, there is a lack of seismicity and tremor downdip beneath Vancouver Island, yet tremor and low-frequency earthquakes (*42*, *43*, *59*) are frequently observed on the JdF side. There is also a clear zone of reduced seismicity between the active Exp tear and its northern slab edge (Fig. 6). We speculate that the Exp tear is continuous between the NFZ and Brooks Peninsula but perhaps is nearly fully torn and mostly aseismic at its northern limit. Unfortunately, the northernmost CASIE21 profile did not extend far enough landward to confirm this hypothesis, but seismicity patterns show that the Exp slab downdip of its tear deepens considerably to the northwest implying an increase in tear maturity toward its slab edge (*42*, *53*). Furthermore, models of migrating slab tears predict that the slab pull force of the torn segment is transferred to the untorn segment, and this dynamic shift causes seaward trench-retreat for the untorn segment and landward trench-advance for the torn segment (*57*, *60*–*62*). The same morpho-tectonic pattern is found in northern Cascadia, where the deformation front locally protrudes seaward on the JdF side and curves landward on the Exp side of the NFZ, respectively (Fig. 3A). This suggests that slab pull gravitational forces of the partially detached downdip Exp slab are no longer supported by the unsubducted Exp oceanic lithosphere and instead have been transferred to the adjacent JdF plate. In addition, SKS shear-wave splitting observations, which are a proxy for mantle flow, reveal a sharp change in the fast-axis orientation across the NFZ. Mantle flow changes from convergence parallel beneath the JdF slab to a counterclockwise rotated orientation across the NFZ and beneath the Exp slab, which progressively becomes more subparallel to PAC-NA translational motion toward Brooks Peninsula (*63*). These patterns argue that mantle flow reorganization beneath the Exp slab has already begun, which further enhances deformation and decoupling of the overriding slab.

**Fig. 10.**
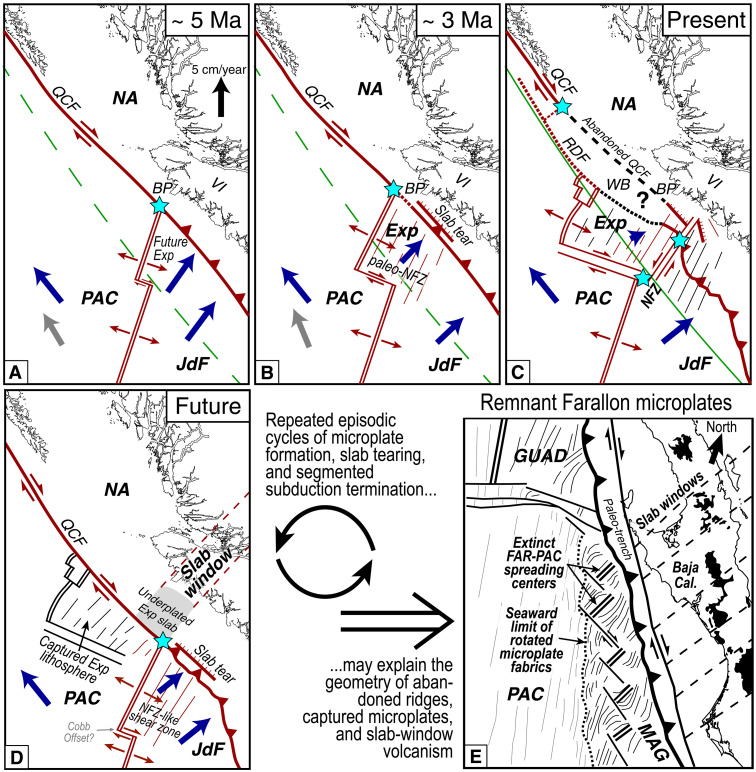
Piecewise plate fragmentation, slab detachment, and segmented subduction termination driven by transform tectonics. (**A** to **D**) Tectonic evolution of subduction termination along the northern Cascadia RTF triple junction at ~5 Ma (A), ~3 Ma (B), present-day (C), and future (D), modified after plate reconstructions of (*26*, *29*, *72*). Blue arrows denote plate motion vectors relative to a fixed NA plate. Red and black lines represent active and inactive structures, respectively. (A) Before creation of the Exp, the JdF, PAC, and NA plates met at a single RTF triple junction near Brooks Peninsula. Blue PAC-NA vector from (*70*); gray PAC-NA vector from (*28*); JdF-NA and future Exp vectors from (*31*). Dashed green line shows PAC-NA small-circle path (*70*). (B) Resistance to subduction causes a westward ridge jump forming the Exp microplate, initiation of left-lateral shearing within the paleo-NFZ, and a trench-parallel slab tear propagating away from the Exp slab edge. Exp-NA vector averaged from Exp-NA vectors at ~5 Ma and present (A and C). Other vectors and green line as in (A). (C) At present, the NFZ forms a tectonic segmentation boundary, fragmenting the incoming plate and decoupling the Exp microplate from adjacent lithosphere through junction with the Exp slab tear. PAC-NA vector from (*70*); JdF-NA and Exp-NA vectors from (*37*). Solid green line shows PAC-NA small-circle path (*70*), which is parallel to the RDF and the most efficient orientation to accommodate PAC-NA transform motion. (D) The future configuration may revert to a single triple junction, where subduction terminates along the Exp domain, resulting in Exp microplate capture and slab-window volcanism. (**E**) Remnant Farallon microplates offshore Baja California, after (*19*), suggesting repeated subduction termination cycles as in [(A) to (D)]. MAG, Magdalena microplate; GUAD, Guadalupe microplate. Black onshore areas outline magnesian andesites and tholeiitic basalts interpreted as slab-window volcanism (*91*).

All these lines of evidence collectively argue for Exp decoupling and impending subduction termination that will eventually lead to a trench-parallel slab window bounded laterally by the Brooks Peninsula and the NFZ, and a narrow trench-perpendicular slab window along the NFZ as it propagates downdip (Figs. 9 and 10). The downdip extent of vertical tearing along the NFZ is unknown; however, the rotated shear-wave splitting orientations and abrupt termination of low-frequency earthquakes and tremor across the NFZ supports its persistence to at least the Johnstone Strait, which separates Vancouver Island from mainland British Columbia. Further landward in the Cascade volcanic arc of British Columbia, distinct Quaternary alkali olivine basaltic lavas of the Wells Gray–Clearwater volcanic field and the Bridge River and Salal Glacier volcanoes of the Garibaldi volcanic belt (Fig. 1A) have been interpreted as evidence of asthenospheric upwelling infilling a vertical slab tear along the NFZ (*64*–*66*). Given the shallow-to-deep slab tearing process documented here, propagation of the NFZ vertical tear to the volcanic arc would require extremely fast tear propagation rates of ~300 km/Myr, which is >3× faster than predicted by geodynamic modeling (*7*). Alternatively, the anomalous volcanism and history of upper-plate deformation (*67*) may instead be related to toroidal flow around the northern slab edge enhanced by decoupling and fragmentation of the slab since initiation of the paleo-NFZ (*68*).

### Implications for subduction termination

The ephemeral creation and isolation of the Exp microplate sheds light on ridge-trench impingement styles of subduction termination, which is likely a common event throughout Earth’s history. The northwestward migration of the RTF triple junction and westward ridge jump to initiate the Exp ridge at ~4 Ma was likely from both local subduction resistance and a broad change in PAC-NA motions due to Yakutat oceanic plateau collision farther north (*26*, *30*–*32*, *69*) (Fig. 10). Concurrently, the NFZ developed within nascent oceanic lithosphere in the wake of the migrating Exp ridge. Since the ridge was highly oblique to the trench, the preexisting Sovanco transform fault offsetting the JdF and Exp ridges (Figs. 1 and 9) was not oriented optimally to accommodate deceleration of Exp beneath NA. Instead, paleo-NFZ shearing exploited favorably oriented abyssal hill faults in warm, weak lithosphere, facilitating clockwise rotation of the Exp microplate as the Exp ridge and RTF triple junction migrated to the northwest. As Exp resisted subduction, slab pull forces initiated a trench-parallel tear within the paleo-NFZ that propagated from the slab edge and buckled the shallow underthrust plate. Strain progressively localized along the current NFZ transform and dissected the migrating slab tear, enabling rapid, multidirectional decoupling of the Exp microplate and eventual breakoff of its slab. The adjacent, severed JdF tear may experience continued seismicity due to intense stresses and downdip viscous coupling of the Exp and JdF slabs beyond the NFZ, but geodetic data suggest that JdF subduction beneath NA is proceeding undisturbed.

Although our data in northern Cascadia provide a snapshot of the dynamic subduction termination process, the identification of a transform-driven slab fragmentation mechanism warrants a re-interpretation of other past examples of subduction termination. Likewise, we can use the final stage spatial architecture and temporal history of ancient subduction termination events to infer the potential future tectonic evolution in northern Cascadia. The most robust comparison for northern Cascadia is the present tectonic morphology of the abandoned microplates offshore Baja California (Fig. 10E), revealed by multibeam bathymetry and magnetic isochron data (*19*). There, abandoned Farallon-PAC ridge segments and microplates show evidence for increasing clockwise rotation from north to south, as the margin progressively transitioned from subduction to transform motion, and slab-window volcanism swept across Baja California. This analog suggests that the present-day complex tectonic configuration in northern Cascadia may evolve in the future, once Exp slab breakoff is complete, to a simpler RTF triple junction setting similar to at ~5 Ma (Fig. 10), where the triple junction will jump back to the southeast when the JdF ridge intersects the trench and the Queen Charlotte Fault re-establishes to the south along the Revere-Dellwood Fault to efficiently accommodate PAC-NA motion (*70*–*72*), enabling capture of the Exp by the PAC plate (*29*). In addition, a trench-parallel slab window will develop where the Exp tear fully detaches, and the subducted slab updip of the Exp tear will be abandoned and fused to NA. The net result would be a lateral shortening of the Cascadia subduction zone by ~75 km, or ~1/12th of its total length. Evidence from Baja California argues that this episodic subduction termination process cyclically repeats, and we speculate that in the future, the northern JdF ridge will again migrate or jump west, perhaps near the existing Cobb Offset, and a new NFZ-like shear zone, Exp-like microplate, and migrating slab tear will develop (Fig. 10).

Our constraints along northern Cascadia capture the 4D nature of slab tearing, i.e., the 3D spatial geometry and inferred temporal evolution, during the final stages of subduction. We propose a subduction termination framework wherein trench-parallel slab tears and transform faults together enable the diachronous fragmentation of oceanic lithosphere and piecewise slab breakoff and microplate capture (Figs. 9 and 10). This segmented, cyclical pattern of microplate formation, slab tearing, and subduction termination modulated by transform faults would result in diachronous, sweeping slab-window volcanism and discrete triple junction jumps over time. The cumulative outcome of this subduction cessation cycle would yield numerous abandoned ridges and microplates, positioned close to the trench, where the microplates become increasingly smaller and rotated, such that the original transform faults offsetting ridge segments align more subparallel to the broader small circle path of transform motion in the RTF triple junction setting. Microplate rotation is likely driven by a combination of progressive reduction in slab pull forces and increased coupling between buoyant young oceanic lithosphere and the overriding plate (*73*). If this subduction termination cycle occurred in northern Cascadia before the ~5 Ma to Present Exp-NFZ-JdF episode, then we would expect to find abandoned microplate fragments on the PAC plate outboard of the Queen Charlotte Fault. There is no obvious bathymetric or geophysical expression of such microplates. It is possible, however, that they exist, but have not been detected because they are obscured by a thick blanket of sediments. Alternatively, the regional tectonic plate motions from ~30 to 5 Ma may have supported a quasi-stable RTF triple junction near Brooks Peninsula, and the substantial change in PAC-NA motion at 5 Ma may have disrupted its stability and initiated the episodic, serial subduction termination process described here and consistent with the Miocene RTF triple junction evolution offshore Baja California.

There are limited geodynamic models of the ridge-trench approach subduction termination process, and the one study that has implemented this scenario in 3D focused on cases where the ridge segments are oriented parallel to the trench and offset by a weak trench-perpendicular transform fault (*7*). In this case, existing transform faults are already oriented optimally to accommodate along-strike decoupling and subduction deceleration, and thus the slab segments may detach rapidly at once, leading to a larger abandoned microplate without the need for internal rotation. This scenario likely applies to the Guadalupe microplate offshore northern Baja California where the fossil PAC-Farallon spreading ridge remains subparallel to the fossil trench (Fig. 10E). Conversely, when the ridge is oriented highly oblique to the trench, existing transform faults are not ideally oriented to facilitate along-strike decoupling, and therefore “NFZ-like” structures are likely to form. These structures are unique and different from typical oceanic transform faults because, although they result in convergence subparallel transform motion, they form in homogenous oceanic lithosphere with no preexisting age or rheological contrasts. Like the NFZ, the initiation of transform motion to accommodate subduction resistance in these ridge-oblique cases may only have preexisting abyssal hill fabrics that are favorably oriented; hence, shearing initiates over a broad region and subsequently localizes once sufficient weakening has taken place. This strain evolution would explain the progressive fragmentation and rotation of microplates, such as the fossil Magdalena microplate offshore southern Baja California. We speculate that NFZ-like structures played a role in the Farallon and other past subduction termination events worldwide, but their ephemeral nature, the lack of geologic record they leave behind, and limited modern in situ examples until this study, might explain why they were not previously identified. NFZ-like transform structures may also help reconcile apparent discrepancies between geologic observations, which often invoke tear initiation at the slab edge and lateral propagation toward the slab interior, and geodynamic models, which predict a wide variety of tearing scenarios but commonly show vertical trench-perpendicular tears in the slab interior (*7*, *57*, *60*, *62*, *74*). The imposed weak transforms and homogenous slab structures in geodynamic models may favor rapid, simultaneous detachment of slab segments, while the heterogenous evolution in northern Cascadia favors lateral trench-parallel tear propagation and prolonged microplate decoupling. More 3D geodynamic models and precise age constraints on slab-window volcanism are needed to further test this proposed framework.

Although individual microplate slabs may detach rapidly, the along-strike impediment of tear propagation by transform faults may explain why ridge trench–type subduction termination is gradual at the broader tectonic plate scale and does not result in major, prompt shifts in plate motions. Conversely, subduction termination via wholesale collision of unsubductable buoyant lithosphere, such as continental blocks and oceanic plateaus, is more likely to trigger sizeable shifts in the balance of broader tectonic forces (*69*, *75*, *76*) and has been thought to even produce favorable conditions for the (re)initiation of subduction (*77*, *78*). While both likely occurred frequently throughout Earth’s history, ridge-trench subduction termination may be more difficult to evaluate for past tectonic events, compared to the buoyant collision style, because the geologic record it leaves behind is more discrete and nuanced. The insights from northern Cascadia and our proposed model for piecewise subduction termination highlights the four-dimensionality of this process and offers a framework to reinterpret the geologic record of slab breakoff and slab-window volcanism for the past Farallon and other subduction cessation events globally. Transform boundaries are ideal candidates to have aided breakup of large oceanic plates into smaller microplates, because they enable along-strike decoupling and efficient tearing of slab segments, allowing discrete triple junction jumps, capturing of microplates, and sweeping slab-window volcanism. Future studies can further test this framework with enhanced geophysical imaging of relict slab fragments, more precise constraints on the internal and bounding structures of abandoned microplates, and improved spatial and temporal mapping of features in the overriding plate, such as slab-window volcanic products and evidence for topographic uplift/subsidence.

## MATERIALS AND METHODS

### Seismic data acquisition and interpretation

MCS reflection data used in this study were acquired during cruise MGL2104 using the R/V *Marcus G. Langseth*. The experiment, CASIE21, consists of 18 primary dip lines, seven primary strike lines, and 16 turn lines spanning from Vancouver Island to the northern Gorda plate along the Cascadia Subduction Zone. The CASIE21 MCS dataset was processed by ION Geophysical using advanced algorithms for preprocessing, prestack depth migration, and postmigration processing to produce high-quality reflection PSDM images. Carbotte *et al.* (*45*) provide an overview on CASIE21 MCS data acquisition and processing. Further details on the CASIE21 data processing routines are described in the ION Geophysical Data Processing Report, which is publicly available along with the raw field data and final PSDM sections from the Marine Geoscience Data System (*44*, *79*).

We analyzed PSDM seismic images along CASIE21 profiles PD02, PD03, PS04, and PS05, which are the primary lines spanning the incoming oceanic lithosphere, the NFZ, and the Exp and JdF slabs along northern Cascadia. All PSDM sections were loaded into the IHS Kingdom Suite software package for interpretation of seismic horizons and faults.

Horizons corresponding with the top of the subducting oceanic crust and plate boundary fault were previously presented by (*45*) for the entire CASIE21 dataset. The top of oceanic crust was identified on the basis of reflection characteristics, including amplitude, frequency, roughness, continuity, and the termination of overlying coherent sediment reflections. The Moho was similarly picked on the basis of reflection characteristics and is generally semicontinuous as a bright, low-frequency reflection at ~6 to 7 km beneath interpreted top of crust. Beneath the accretionary prism, the plate boundary interface is identified on the basis of the geometry and depth extent of faults transecting sediment packages in the overlying accretionary prism, as well as other characteristics described in (*45*). Offshore Vancouver Island, the frontal prism thrust faults shoal into a décollement near the top of the subducting oceanic crust; hence, the plate boundary fault and top of the oceanic crust are interpreted as the same horizon. The subducting top of the crust and oceanic Moho display similar reflectivity characteristics beneath the prism as outboard of the trench, although the amplitudes are typically weaker and more variable.

In addition to horizon picking, we also interpreted major faults along the seismic profiles to carry out a structural analysis. Faults in the sediment section were identified from offset sediment horizons. Sediment faults have an average dip of ~60° (Fig. 6) and generally exhibit apparent normal offsets. Some sediment faults dip more steeply (70° to 90°), and differential deformation patterns in sediment packages across the fault plane suggest strike-slip motion. It is difficult to quantify the amount of strike-slip versus normal faulting from 2D seismic alone; however, steeper-dipping sediment faults were more abundant within the NFZ, which is consistent with focal mechanism patterns suggesting more oblique faulting concentrated here.

Within the crust and mantle, the deep-penetrating seismic images revealed a cross-cutting network of unusually bright dipping reflectors (Figs. 3 and 5). These reflections have apparent dips ranging from 20° to 80° (Fig. 6), are generally semicontinuous from the crust to ~10 km into the mantle and have listric geometries curving to shallower dips with increasing depth. Several key pieces of evidence suggest that these reflections arise from seismic impedance contrasts within faults and thus represent fault-plane reflections. First, some of these reflections can be traced upward to clear offsets in the overlying sediment section, especially within the active NFZ. For example, the NNF fault on profile PD03 is clearly visible from a seafloor scarp, offset sediments, a large basement offset, and a bright fault-plane reflection extending through the crust and into the upper mantle (fig. S2). Second, these reflections are often offset and kinked where they cross other bright reflectors, which we interpret as fault-plane reflections that were cut and deformed by multiple generations of anastomosing faults. These faults do not generally have large displacements of the top of crust and Moho, especially outside of the NFZ. However, minor offsets in the Moho reflection occur where the reflections transect the crust into the mantle, further supporting that dipping reflectors arise from fault-planes in the subsurface. On the seaward end of PS04, the dipping reflectors cause an en-echelon stepping pattern in the Moho reflection (fig. S3), which is suggestive of a pervasive shearing fabric as documented in outcrop analogs. Our interpretation strategy was to pick all bright and semi-continuous reflections to view the general architecture of subsurface fault networks and to not overinterpret the seismic sections. The anastomosing shear network indicates multiple generations of faults preserved where one fault may become inactive and then a different fault becomes active and cuts across previous fault planes.

The fault network imaged within the incoming oceanic crust and upper mantle of the NFZ region is abnormally bright and coherent compared with past studies in other areas. Faults within oceanic crust and mantle typically do not produce obvious fault-plane reflections in seismic data but are rather interpreted to be present based on offsets in overlying sediments, and top of oceanic crust, or the Moho reflection. The amplitude of these deep reflections is variable on our profiles, with the brightest and most continuous reflectors observed on PS04 and weakest on PS05 (Fig. 5 and figs. S1 to S4).

### Geometry analysis of paleo- and active NFZ faults

We carried out a geometry analysis of the deep crust and mantle reflectors to understand their tectonic origin (Fig. 6). Our 2D seismic dataset allows us to constrain the apparent dip of these faults by measuring the average dip along individual fault-plane reflections, and differences for each profile reflect variable orientation of the seismic profiles with respect to the dominant fault trends. In some cases, where the same fault-plane reflection can be confidently correlated on multiple profiles, we use the apparent dips and geometry of our profiles to calculate true dips. This exercise was only possible on the primary strands of the NFZ, which could be confidently associated on multiple profiles because of their obvious structural characteristics and closely spaced line crossings. For example, the NNF fault has an apparent dip of 44° on PS04 and 50° on PD03. On the basis of these measurements, and an internal angle of 103° between the profiles, we calculate that the NNF has a true strike of ~34° clockwise from the strike of PS04, or N27°E. This estimate agrees very well with the NNF strike of N28°E mapped from multibeam bathymetry data of its seafloor scarp (Fig. 2).

Because of the limited spatial nature of our 2D dataset, we cannot directly calculate true strike and dips for other interpreted faults. However, previous studies have argued that faulting in this region, and in other subduction zone settings, takes advantage of weak preexisting structural features within oceanic crust, most notably abyssal hill normal faults formed at the ridge axis (*80*). The active NNF and SNF faults strike within ~10° of the dominant magnetic isochron trend, which is consistent with the expected orientation of Riedel Shears in a left-lateral shear zone with a dominant shear orientation of ~N42°E (Fig. 6E). For simplicity, if we assume the other faults have similar orientations, we can estimate true dips from their measured apparent dips, which vary substantially between the four profiles. As a result, the calculated true dips for all profiles exhibit similar normal distributions centered around ~60° (Fig. 6C). The similarity in these distributions implies that magnetic-isochron-subparallel strikes are most probable for the deep faults. This orientation is also most consistent with variations in reflectivity between the profiles, which are brightest on PS04 where apparent dips are shallowest. Our geometric analysis implies paleo-NFZ and modern NFZ faults strike subparallel to magnetic isochrons. This orientation, along with estimated true dips of ~60° and limited top of crust and Moho offsets (outside of the active NFZ), allow us to decipher the origin and nature of these faults, which we suggest represent the reactivation of inherited abyssal hill normal faults formed at the ridge by left-lateral transtensional shearing associated with initiation of the NFZ during resistance to subduction.

### Synthesis with earthquake catalogs

We integrated our seismic images with recently published earthquake hypocenter and focal mechanism catalogs (*40*–*43*) to help characterize subsurface structure, the location of active deformation, and spatial variations in the stress state along the margin. These passive-source studies (*40*–*43*) analyzed waveforms from nearby permanent and temporary networks (i.e., Canadian National Seismographic Network, NEPTUNE, POLARIS, and SeaJade I and II) and implemented earthquake detection and joint hypocenter-velocity inversions using TomoDD software. Because each study used different inversion parameters, station distributions, and velocity models, the final relocated events have different hypocenter locations and depths. Therefore, we considered all catalogs in the synthesis with our seismic images despite some duplicate earthquake events in the catalogs. We integrated the final hypocenters (2209 events) from (*43*), the final hypocenters (3918 events) and “A” class focal mechanisms (1089 solutions) from (*40*), the “S” class hypocenters (8685 events) from (*42*), hypocenters (18,441 events) from (*41*), and focal mechanisms (1089 events) for larger events from the Canadian National Earthquake Database (*46*). See corresponding articles and the Supplementary Materials for more information on these studies and their earthquake catalogs.

We plotted the hypocenters and focal mechanisms from all five catalogs in map view using Generic Mapping Tools (GMT) software (*81*) to determine spatial patterns of deformation and correlations with our seismic images. We also projected earthquake hypocenters and focal mechanisms into the plane of our seismic profiles to inspect the relationship between seismicity and fault structures imaged in our dataset. Focal mechanisms in map view are plotted as lower hemisphere projections and in cross sections are plotted as back hemisphere projections. Focal mechanisms were classified by slip-type using the FMC software (*82*). Hypocenter events up to ±15 km away from the 2D plane of our seismic profiles were included. We only projected hypocenter events from the (*40*, *42*, *43*) catalogs onto the seismic images. We included epicenter locations from the (*41*) catalog in map view but not projected hypocenters because the depths of these events were fixed to 5 km. For simplicity, we only show projected hypocenters and focal mechanisms from the catalog of (*40*) on PSDM sections in Fig. 3, since the high-resolution methodology of this study resulted in the most accurate relocations that best match our seismic reflection images. Figure 8 and figs. S1 to S4 show Merrill *et al.* (*42*) hypocenters (purple), Savard *et al.* (*43*) hypocenters (blue), and Hutchinson *et al.* (*40*) hypocenters (pink) and focal mechanisms down to 45 km depth. Comparison between the catalogs generally shows similar patterns, although events in (*42*) are located deeper than those in (*40*), especially beneath the prism (e.g., fig. S1). Hypocenters from (*42*) also tend to locate slightly west of the (*40*) events (Fig. 7).

For the four earthquake catalogs (*40*–*43*), hypocenters before TomoDD have nominal major, minor, and depth error ellipsoids of ~3.5, 1.5, and 7 km, respectively. After TomoDD, these errors are substantially reduced, in particular for events within the NFZ. However, since these earthquake catalogs have different hypocenter depths beneath the accretionary prism (±5 km), but their epicenter locations are generally better resolved (±1 km), we analyzed the lateral density of earthquakes along each seismic profile. For this analysis, we included epicenters from (*40*, *42*, *43*) catalogs. We note that there are overlapping events between the catalogs, which will lead to duplicate counts toward earthquake density; however, the primary goal of this exercise is to qualitatively show the correlation between regions of high earthquake activity with structural features imaged in the seismic profiles. This lends confidence in our interpretation of which structures may be active or not. Earthquake density was calculated in rolling 5-km-wide bins every 1 km along each profile using GMT (*81*). We then plotted the binned density on a log_10_ scale between 1.5 and 3.0 (~32 to 3000 events per 5 km), since high levels of seismicity in the NFZ would saturate the results on a linear scale, and using a logarithmic scale minimizes the inclusion of some duplicative events. The resulting earthquake density is shown along the bottom of the seismic sections (Figs. 3, 5, and 8 and figs. S1 to S4) and highlights areas of active deformation along the profiles.

The recent passive-seismic studies mentioned above revealed several trench-parallel lineations of seismicity landward of the trench and proposed various interpretations for their tectonic origin which we re-evaluate considering the new CASIE21 seismic images of the slab structure. The western lineation, referred to as the “Nootka Sequence Fault (NSF)” by (*40*) and “2014 cluster” by (*42*), lies on the Exp side of the NFZ and experienced a 2014 *M*_w_ 6.6 earthquake and aftershock sequence that was recorded by the SeaJade experiment. The NSF is ~35 km long and comprises a narrow, near vertical fault zone, extending from approximately ~6 to 40 km depth (fig. S1). Seismicity on the NSF broadens with depth starting at ~25 km and hence indicates a potential widening of the fault zone into the mantle lithosphere. The depth uncertainty of seismicity has led to a debate about whether the NSF is solely within the subducting oceanic plate or also ruptures the overriding plate. For instance, the 2014 *M*_w_ 6.6 Centroid Moment Tensor solution is ~20 km shallower compared to the hypocenter locations determined by (*40*, *42*). In our seismic images, the shallow earthquakes begin near the top of the subducting slab and thus imply that the NSF represents a major fault zone solely within the Exp slab. Hutchinson *et al.* (*53*) found a downward deflection in the slab to the northwest along the NSF and suggested that this may reflect a bend or tear in the Exp slab aligned perpendicular to the direction of subduction. Further, tomography models from both (*42*) and (*40*) found slower velocities just NE of the NSF, which can be explained by the drop in the Exp slab imaged in our data. Collectively, our images showing a major offset in the slab colocated with extensive normal faulting focal mechanisms and near vertical seismicity elongated parallel to the trench confirm that the NSF represents a tear in the Exp slab.

The eastern lineation (EL), referred to as the “eastern lineation” by (*40*), “F1” by (*43*), and “2004 and 2011 clusters” by (*42*), lies on the JdF side of the NFZ and ruptured in notable 2004 *M*_w_ 6.3 and 2011 *M*_w_ 6.4 earthquakes with associated aftershocks. The EL is similarly ~35 km long but has a more northerly strike compared to the NSF but still is oriented parallel to the nearby trench. A near vertical band of seismicity is present on the EL from ~15 to 40 km depth but appears wider compared to the NSF (fig. S2). These events are nearly all within the oceanic crust and mantle, suggesting that the EL fault zone is localized to the JdF slab. Further landward of the EL, another notable lineation termed “F2” by (*43*) forms a similar steep band of seismicity. Seismicity outlining the subducting slab appears to steepen downdip of EL but flatten near the F2 lineation. Unfortunately, our seismic image along PD03 does not extend to F2, but the inferred slab bend geometry by (*43*) is consistent with the slab bend we image at EL. Our seismic imaging of this buckling feature colocated with normal faulting focal mechanisms and steep seismicity elongated parallel to the trench suggests that the EL represents a nascent tear in the JdF slab at a more immature stage compared to the NSF. It is also possible that we do not image a sharp slab offset along the EL because PD03 crosses at the southernmost edge of the fault zone. Here, the projected seismicity appears slightly downdip of the apex of the slab bend likely because of the oblique orientation at which PD03 crosses the southernmost tear edge. Savard *et al.* (*43*) show a cross section through the center of the JdF tear, where the seismicity is concentrated at the inferred apex of the JdF slab buckle. Regardless, the lack of seismicity and tremor downdip of the Exp tear but presence of these features on the JdF side further supports that the Exp slab tear represents a more mature stage of decoupling from its deeper slab.
