# Supplementary material for: Slab tearing and segmented subduction termination driven by transform tectonics
Source: Sci Adv. 2025 Sep 24;11(39):eady8347. doi: 10.1126/sciadv.ady8347 (PMC12459466; doi:10.1126/sciadv.ady8347)
Supplement: Supplementary file 1 — Figs. S1 to S4 [file sciadv.ady8347_sm.pdf]

Science Advances
AAAS

Supplementary Materials for

Slab tearing and segmented subduction termination driven by transform tectonics

Brandon Shuck *et al.*

Corresponding author: Brandon Shuck, bshuck@lsu.edu

Sci. Adv. **11**, eady8347 (2025)
DOI: 10.1126/sciadv.ady8347

This PDF file includes:

Figs. S1 to S4

Fig. S1.

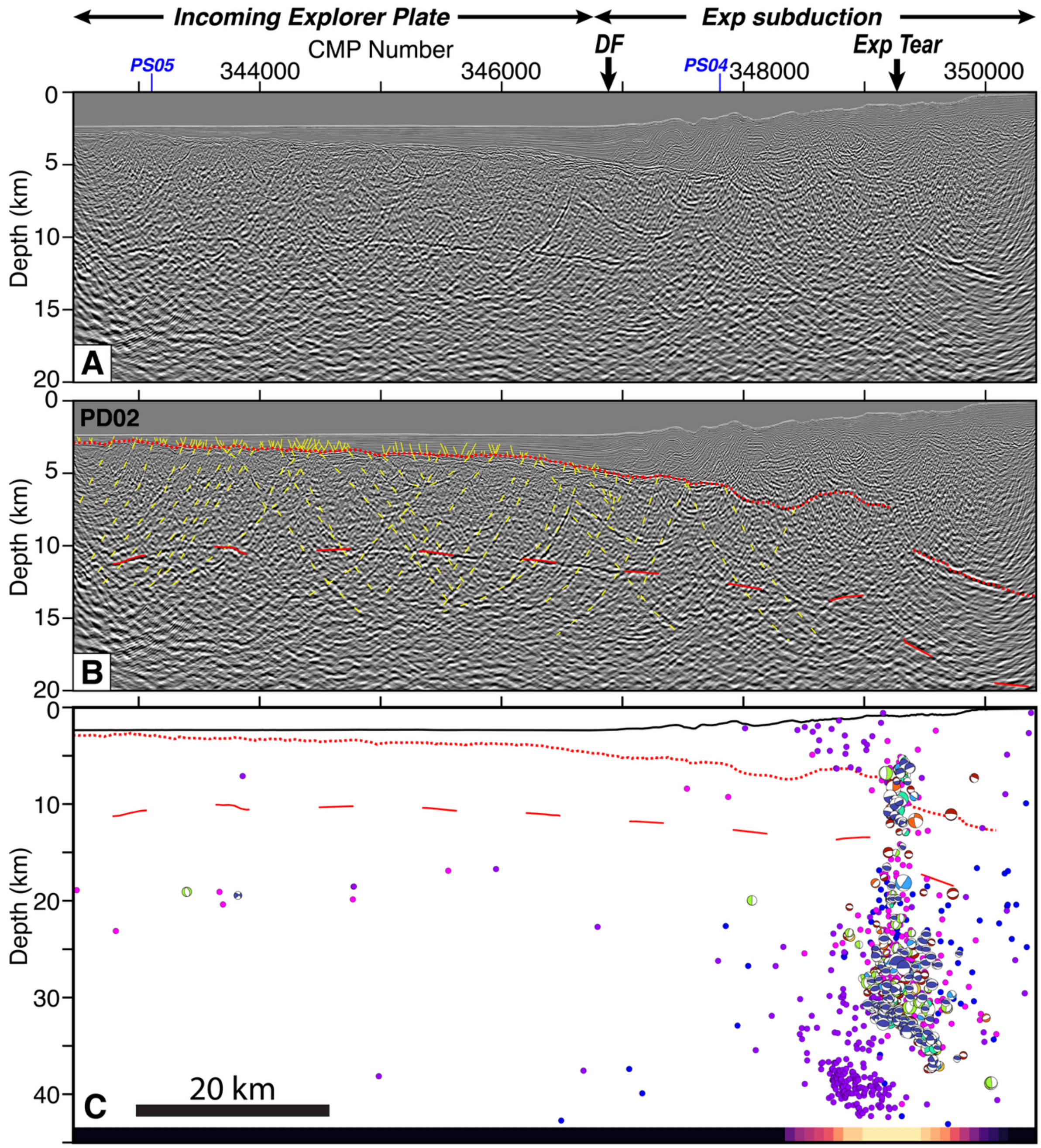

Fig. S1. Seismic reflection image and projected seismicity on profile PD02 spanning the incoming Explorer (Exp) microplate and Exp slab. Panels **A** and **B** are shown at VE = 1.5, panel **C** at VE = 1. **(A)** Uninterpreted pre-stack depth migrated seismic reflection image. DF = Deformation Front. **(B)** Interpreted seismic section, same as shown in Fig. 3B. **(C)** Earthquake hypocenters from ref (*40*) in pink, ref (*42*) in purple, and ref (*43*) in blue, and focal mechanisms from ref (*40*) are projected +/- 15 km into the profile. Earthquake density is shown along the base of the section as in Fig. 3. Seismicity is narrowly focused into a vertical band that is spatially coincident with a slab offset in the seismic image, consistent with tearing and ongoing detachment of the Exp slab at this location.

Fig. S2.

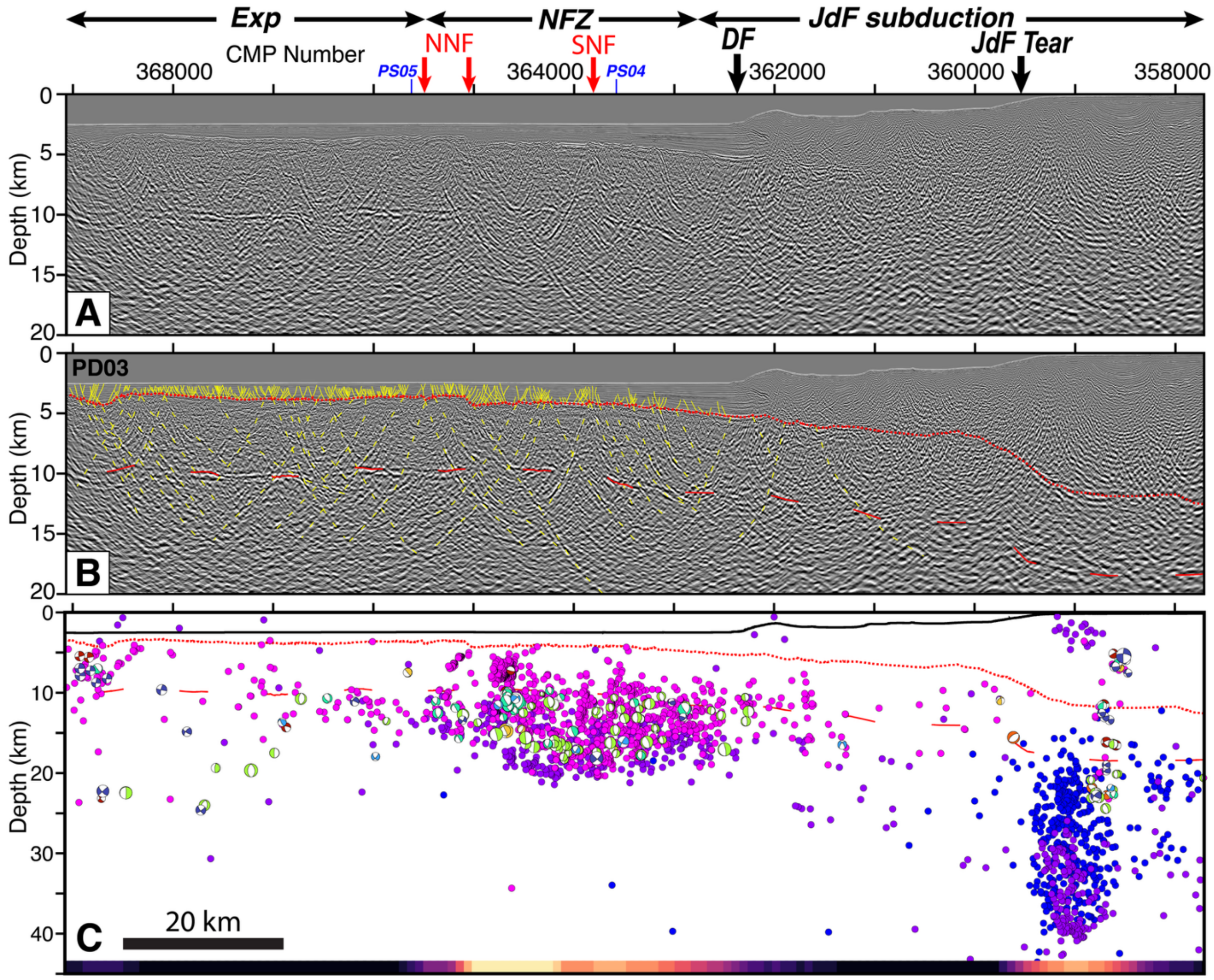

Fig. S2. Seismic reflection image and projected seismicity on profile PD03 spanning the incoming Explorer (Exp) microplate, the Nootka Fault Zone (NFZ), and Juan de Fuca (JdF) slab. Panels **A** and **B** are shown at VE = 1.5, panel **C** at VE = 1. **(A)** Uninterpreted pre-stack depth migrated seismic reflection image. NNF = Northern Nootka Fault; SNF = Southern Nootka Fault; DF = Deformation Front. **(B)** Interpreted seismic section, same as shown in Fig. 3C. **(C)** Earthquake hypocenters and focal mechanisms are shown as in Fig. S1. Earthquake density along the base of the section is shown as in Fig. 3. Seismicity occurs in a near-vertical band, but less abundant and broader than on PD02, and is spatially coincident with a bend in the slab in the seismic image, consistent with less-developed tearing of the JdF slab at this location. The wider band of vertical seismicity along the JdF tear is focused near the base of the slab buckle, which is likely because PD03 crosses at the southernmost edge of the JdF tear and the hypocenters are projected into the oblique plane of our profile. Ref (*43*) shows a cross section through the center of the JdF tear, where the vertical band of seismicity appears coincident with the apex of the slab buckle.

Fig. S3.

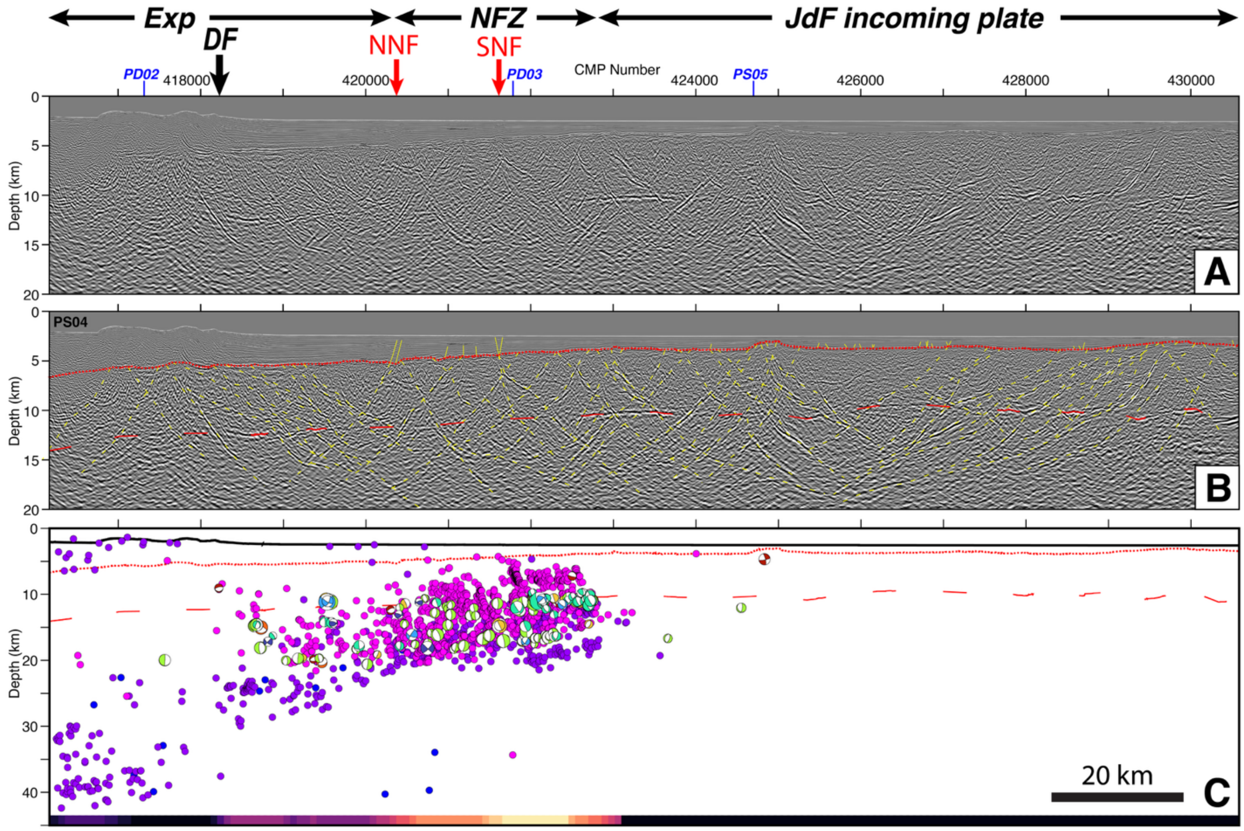

Fig. S3. Seismic reflection image and projected seismicity on profile PS04 spanning the Juan de Fuca (JdF) incoming plate, the Nootka Fault Zone (NFZ), and Explorer (Exp) slab. Panels **A** and **B** are shown at VE = 1.5, panel **C** at VE = 1. **(A)** Uninterpreted pre-stack depth migrated seismic reflection image. NNF = Northern Nootka Fault; SNF = Southern Nootka Fault; DF = Deformation Front. **(B)** Interpreted seismic section, same as shown in Fig. 3D. **(C)** Earthquake hypocenters and focal mechanisms are shown as in Fig. S1. Earthquake density along the base of the section is shown as in Fig. 3. Bright crust-mantle reflections reveal a pervasive anastomosing shear fabric associated with paleo-NFZ and active NFZ faults. Note the diminished Moho reflectivity within the modern NFZ where earthquakes are concentrated in the crust and mantle.

Fig. S4.

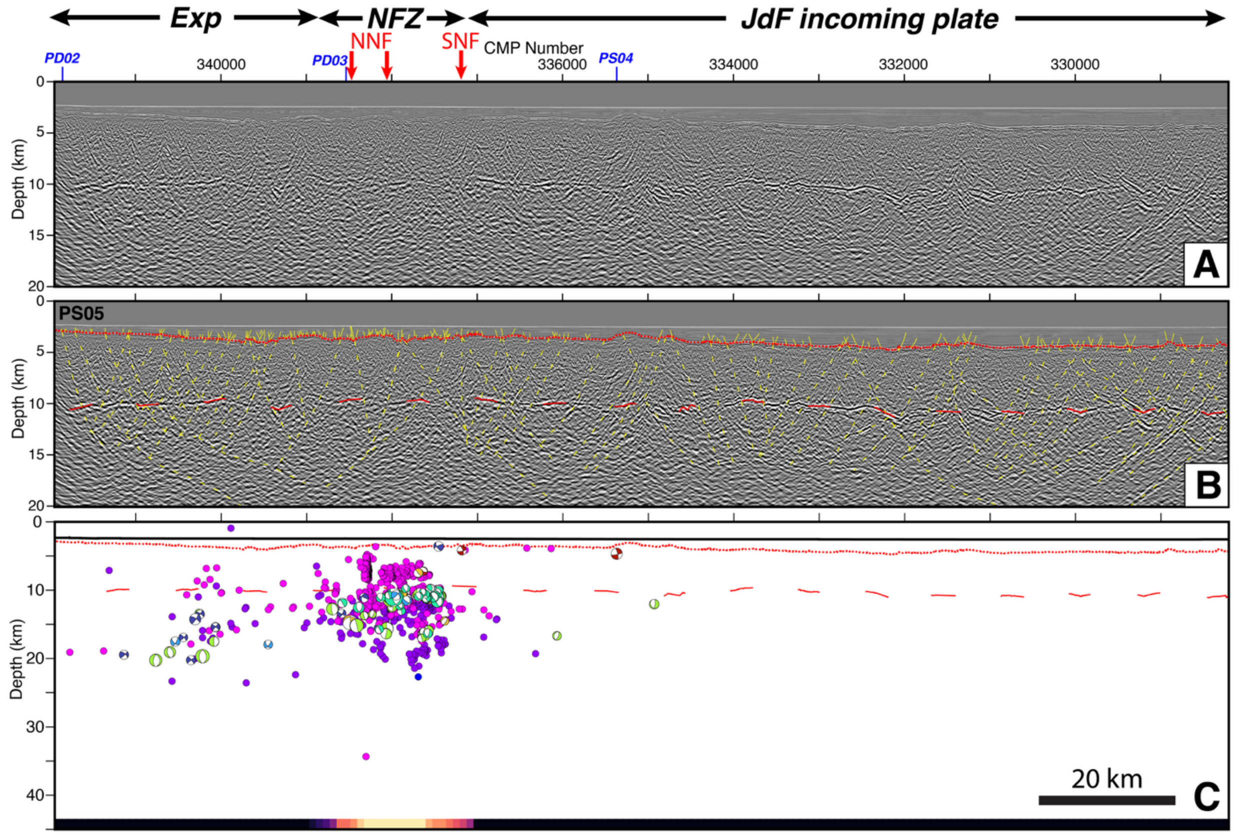

Fig. S4. Seismic reflection image and projected seismicity on profile PS05 spanning the incoming Juan de Fuca (JdF) lithosphere, the Nootka Fault Zone (NFZ), and the incoming Explorer (Exp) lithosphere. Panels **A** and **B** are shown at VE = 1.5, panel **C** at VE = 1. **(A)** Uninterpreted pre-stack depth migrated seismic reflection image. NNF = Northern Nootka Fault; SNF = Southern Nootka Fault. **(B)** Interpreted seismic section, same as shown in Fig. 3E. **(C)** Earthquake hypocenters and focal mechanisms are shown as in Fig. S1. Earthquake density along the base of the section is shown as in Fig. 3. PS05 crosses perpendicular to the NFZ and hence the fault zone appears narrower (~20 km wide) than on other profiles. Note the diminished Moho reflectivity within the modern NFZ.
